# Estimating the Multidimensional Generalized Graded Unfolding Model with Covariates Using a Bayesian Approach

**DOI:** 10.3390/jintelligence11080163

**Published:** 2023-08-14

**Authors:** Naidan Tu, Bo Zhang, Lawrence Angrave, Tianjun Sun, Mathew Neuman

**Affiliations:** 1 Department of Psychology, University of South Florida, Tampa, FL 33620, USA; 2 School of Labor and Employment Relations & Department of Psychology, University of Illinois Urbana-Champaign, Champaign, IL 61820, USA; 3 Department of Computer Science, University of Illinois Urbana-Champaign, Urbana, IL 61801, USA; 4 Department of Psychological Sciences, Kansas State University, Manhattan, KS 66506, USA; 5 Department of Psychological & Brain Sciences, Texas A & M University, College Station, TX 77840, USA

**Keywords:** multidimensional generalized graded unfolding model (MGGUM), item response theory (IRT), Bayesian estimation, covariates

## Abstract

Noncognitive constructs are commonly assessed in educational and organizational research. They are often measured by summing scores across items, which implicitly assumes a dominance item response process. However, research has shown that the unfolding response process may better characterize how people respond to noncognitive items. The Generalized Graded Unfolding Model (GGUM) representing the unfolding response process has therefore become increasingly popular. However, the current implementation of the GGUM is limited to unidimensional cases, while most noncognitive constructs are multidimensional. Fitting a unidimensional GGUM separately for each dimension and ignoring the multidimensional nature of noncognitive data may result in suboptimal parameter estimation. Recently, an R package *bmggum* was developed that enables the estimation of the Multidimensional Generalized Graded Unfolding Model (MGGUM) with covariates using a Bayesian algorithm. However, no simulation evidence is available to support the accuracy of the Bayesian algorithm implemented in *bmggum*. In this research, two simulation studies were conducted to examine the performance of *bmggum*. Results showed that *bmggum* can estimate MGGUM parameters accurately, and that multidimensional estimation and incorporating relevant covariates into the estimation process improved estimation accuracy. The effectiveness of two Bayesian model selection indices, WAIC and LOO, were also investigated and found to be satisfactory for model selection. Empirical data were used to demonstrate the use of *bmggum* and its performance was compared with three other GGUM software programs: *GGUM2004*, *GGUM*, and *mirt*.

## 1. Introduction

Item Response Theory (IRT) plays an important role in organizational research (Cai et al. 2016; Lang and Tay 2021; Nye et al. 2020; Reise and Waller 2009). Out of dozens of IRT models, the unfolding family has gained increasing attention in the past decade among researchers. Unlike dominance IRT models (e.g., Rasch Model, Graded Response Model) that assume a monotonical relationship between a respondent’s latent trait level and the probability of endorsing an item, unfolding models assume that the probability of endorsing an item is inversely related to the distance between a respondent’s latent trait level and the item location, such that the closer they are, the more likely the respondent will endorse the item (Drasgow et al. 2010). Undoubtedly, the Generalized Graded Unfolding Model (GGUM; Roberts et al. 2000) is the most popular model within the family of unfolding models, largely due to the availability of the freely accessible software *GGUM2004* (Roberts et al. 2006). Compared to dominance models, the GGUM has been shown to better represent how individuals respond to items measuring noncognitive constructs such as personality (Cao et al. 2015; Carter et al. 2014; Zhang et al. 2020), vocational interests (Tay et al. 2009), emotional intelligence (Cho et al. 2015), job satisfaction (Carter and Dalal 2010), and adult attachment styles (Sun et al. 2021). Failing to adopt an appropriate IRT model for noncognitive data may result in the selection of unqualified candidates (Stark et al. 2006). Additionally, it may lead to reduced criterion-related validity (Sun et al. 2021) or low power to detect curvilinear effects if they exist in the population (Cao et al. 2018; Carter et al. 2017).

Despite the increasing popularity of the GGUM, research on how to better utilize the model to produce more accurate person and item parameter estimates is still needed. Specifically, researchers are still constrained by the following three issues: (1) extreme item parameter and standard error estimates, (2) inability to estimate the multidimensional GGUM (MGGUM), and (3) inability to incorporate covariates into the estimation. Regarding the first issue, *GGUM2004* often produces extreme item location parameters (e.g., δ = 30) and huge standard errors (e.g., SE = 100). These extreme estimates will jeopardize the quality of the final item set when the GGUM is used for scale development. They may also mask differential item functioning and hamper the accuracy of trait score estimates, resulting in biased assessment of individuals and unfair selection outcomes. When it comes to the second issue, it is well-known in the dominance IRT literature that simultaneous estimation of a multidimensional scale would lead to more accurate parameter estimates than separate unidimensional estimation for each subscale (Wang et al. 2004). It also provides more accurate estimates of the correlations between traits, particularly when the subscales are short (Hinkin 1995). However, most if not all applications of the GGUM have been limited to unidimensional estimation because *GGUM2004* can only fit unidimensional models. Given the importance of accurately estimating item parameters (e.g., for computerized adaptive testing), trait scores (e.g., for personnel selection), and correlations between traits (e.g., for studying the relationship among traits), the MGGUM is clearly a desirable extension. As for the third issue, currently, when researchers are interested in the relationships between traits and covariates (e.g., gender difference in conscientiousness), they have to first obtain the trait scores and use these scores to estimate the difference, which is often downward biased because of measurement error. If covariates can be incorporated into the estimation process, researchers can easily obtain bias-free estimates of the relationships between covariates and traits because measurement error can be accounted for. Moreover, both the dominance IRT literature and the unidimensional unfolding IRT literature have shown that the incorporation of covariates (e.g., age, gender, education) into the estimation process can improve the estimation accuracy of trait scores (Curran et al. 2016; Joo et al. 2022; Usami 2011), especially in cases of missing data or short scales (Thomas 2002). We would expect similar benefits for the MGGUM.

This research evaluates the effectiveness of a Bayesian estimation algorithm implemented in the R package *bmggum* (Tu et al. 2021) in addressing the three issues discussed above. Although *bmggum* (Tu et al. 2021) has been available on CRAN for over two years, no simulation evidence exists yet to support the accuracy of the Bayesian estimation algorithm implemented by it. In addition, while *bmggum* provides two Bayesian model fit diagnostics (WAIC and LOO; Vehtari et al. 2017) for model selection, it is unknown how accurate these two model fit indices are for selecting the data-generating model. Therefore, the second aim of this research is to examine the power of the two model fit indices for model selection. A step-by-step tutorial was also provided in Appendix A of the Supplementary Material on how to fit MGGUM with covariates in R using the *bmggum* package. Overall, our goal is to provide interested readers with simulation evidence to support the use of MGGUM with covariates for noncognitive assessment.

### 1.1. The GGUM and Its Multidimensional Extension

Unlike dominance models, in which the probability of endorsing an item is monotonically related to respondents’ latent trait levels, in the GGUM (Roberts et al. 2000), the distance between the item location and the trait levels of respondents is key to endorsing an item. Respondents are more likely to disagree with an item if their trait levels are higher or lower than the trait level indicated by the item, and they are more likely to agree with an item if their trait levels are similar to the trait level indicated by the item. The probability of endorsing a particular response option for an item in the GGUM is mathematically defined as

(1) $$P[Z_{i}=z\;|\;\theta_{j}]=\frac{\exp\{\alpha_{i}[z\left(\theta_{j}-\delta_{i}\right)-\sum_{k=0}^{z}\tau_{\mathrm{ik}}]\}+\exp\left\{\alpha_{i}[(M-z)\left(\theta_{j}-\delta_{i}\right)-\sum_{k=0}^{z}\tau_{ik}]\right\}}{\sum_{w=0}^{C}\left\{\exp\{\alpha_{i}[w\left(\theta_{j}-\delta_{i}\right)-\sum_{k=0}^{w}\tau_{\mathrm{ik}}]\}+\exp\left\{\alpha_{i}[(M-w)\left(\theta_{j}-\delta_{i}\right)-\sum_{k=0}^{w}\tau_{ik}]\right\}\right\}}$$

where θ_j_ is the latent trait level of person j, α_i_ is the discrimination parameter of item i, δ_i_ is the location parameter of item i, τ_ik_ is the kth subjective response category threshold for item i, C is the number of response options minus 1, and M = 2C + 1.

The widely used GGUM is currently limited to unidimensional cases. When it is used for noncognitive constructs that are often multidimensional in nature (e.g., personality, vocational interest), a common practice is to fit a unidimensional GGUM separately for each dimension, given that most existing programs (e.g., *GGUM2004*) were developed only for the unidimensional GGUM. However, this approach ignores the nonzero correlations between dimensions that could be leveraged to improve parameter estimation accuracy. Ideally, researchers would fit an MGGUM to multidimensional data so that all dimensions can be estimated simultaneously. This is less cumbersome and, more importantly, takes advantage of the correlations between dimensions to improve estimation efficiency (Wang et al. 2004). Simultaneously estimating all dimensions allows for the utilization of information from correlated dimensions to enhance the estimation accuracy of each dimension, particularly when the tests are short and the correlations among dimensions are substantial. In real testing situations, tests are often too short to provide adequate measurement accuracy for individuals because multiple traits need to be assessed within a limited time frame. With multidimensional estimation, trait scores can be estimated with higher accuracy (Wang et al. 2004). The correlations between dimensions, which in themselves are also core aspects of construct validity, can also be estimated directly and more accurately when multiple dimensions are estimated simultaneously (Wang et al. 2004). This is beneficial when researchers and practitioners are interested in the relationships between traits (e.g., conscientiousness and job satisfaction). Instead of correlating the fallible estimated trait scores obtained from several unidimensional GGUMs, the MGGUM can be applied to directly estimate the variance–covariance matrix of the traits, resulting in more accurate estimates of the correlations between traits.

**Figure 1:**
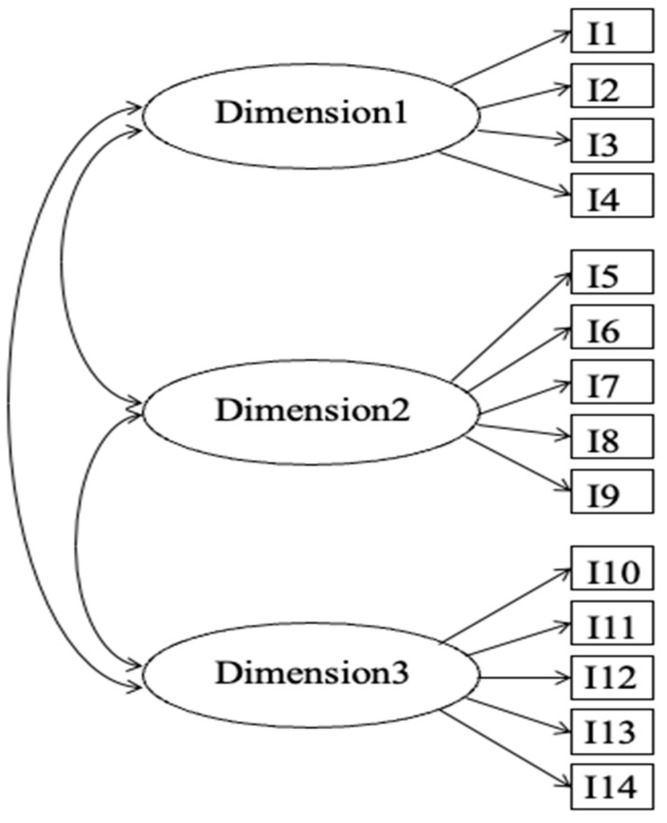
An example of between-item dimensionality where each item only loads on one dimension.

In the MGGUM, the probability of endorsing a certain response option of an item is given by

(2) $$\begin{array}{c} P[Z_{ij}=z|\theta_{j}]=\frac{\exp\left[\left(z\sqrt{\sum_{d=1}^{D}\alpha_{id}^{2}{\left(\theta_{jd}-\delta_{id}\right)}^{2}}\right)-\sum_{k=0}^{z}\psi_{ik}\right]+\exp\left[\left((M-z)\sqrt{\sum_{d=1}^{D}\alpha_{id}^{2}{\left(\theta_{jd}-\delta_{id}\right)}^{2}}\right)-\sum_{k=0}^{z}\psi_{ik}\right]}{\sum_{w=0}^{C}\left(\exp\left[\left(w\sqrt{\sum_{d=1}^{D}\alpha_{id}^{2}{\left(\theta_{jd}-\delta_{id}\right)}^{2}}\right)-\sum_{k=0}^{w}\psi_{ik}\right]+\exp\left[\left((M-w)\sqrt{\sum_{d=1}^{D}\alpha_{id}^{2}{\left(\theta_{jd}-\delta_{id}\right)}^{2}}\right)-\sum_{k=0}^{w}\psi_{ik}\right]\right)},\\ \mathrm{with}\;\psi_{\mathrm{ik}}=\sum_{d=1}^{D}\alpha_{id}\tau_{ik} \end{array}$$

where θ_jd_ is the latent trait level of person j on the d^th^ dimension, and **θ**_j_ = (θ_j1_, θ_j2_, …, θ_jD_) are assumed to follow a multivariate normal distribution. α_id_ is the discrimination parameter of item i on the d^th^ dimension, δ_id_ is the location parameter of item i on the d^th^ dimension, ψ_ik_ is the threshold parameter of the k^th^ multidimensional subjective response category for item i, and τ_ik_ is the k^th^ subjective response category threshold for item i. D is the number of dimensions. C is the number of response options minus 1 and M = 2C + 1. The MGGUM estimated in the package *bmggum* only considers between-item multidimensionality (simple structure), which means that each item measures only a single trait, as shown in Figure 1. Therefore, α_id_, δ_id_, and τ_ik_ = 0 for all d except one. Within each dimension, the unidimensional GGUM still applies. Users will need to rely on theories to decide which item loads on which factor, just like in confirmatory factor analysis. Note that the MGGUM defined in Equation (2) resembles the confirmatory multidimensional generalized graded unfolding model (CMGGUM) proposed in Wang and Wu (2016). The CMGGUM was proposed to handle both between and within-item multidimensionality, which means that it allows cross-loadings. In the case of between-item dimensionality, MGGUM is equivalent to CMGGUM.

### 1.2. Estimating the MGGUM

The MGGUM is a complex model with a functional form that includes multiple freely estimated parameters. For unidimensional GGUM estimation, both the software *GGUM2004* and the R package *GGUM* (Tendeiro and Castro-Alvarez 2019) estimate item parameters using marginal maximum likelihood (MML). MML requires deriving first and second derivatives of likelihood functions, matrix inversion, and multidimensional integration, which makes it less suitable for complex models with three or more dimensions. It also often produces extreme estimates of standard errors, especially for items with extreme location parameters. Markov Chain Monte Carlo (MCMC) is an alternative estimation method that is deemed more appropriate for complex high-dimensional IRT models and has been found to provide more reasonable estimates of standard errors and comparable item parameter estimation accuracy to MML (de la Torre et al. 2006; Lee et al. 2019). However, previous applications of MCMC to GGUM have only focused on the unidimensional model. The R package *bmggum* (Tu et al. 2021) extends the MCMC estimation to MGGUM. Specifically, the *bmggum* package uses the state-of-the-art Hamiltonian Monte Carlo (HMC) algorithm in Stan (Stan Development Team 2020) as the backend estimation engine. HMC is an MCMC sampling algorithm that is considered more efficient than other commonly used MCMC sampling algorithms (e.g., Gibbs sampling and Metropolis–Hastings algorithm), and thus reduces the time it takes to estimate parameters (Luo and Jiao 2018). However, no evidence is available yet to support the effectiveness of the estimation algorithm implemented in *bmggum*. Therefore, this research evaluates the performance of the algorithm implemented in *bmggum* in estimating MGGUM. In the package *bmggum*, the likelihood function of the MGGUM response data given all model parameters is given by

(3) $$P\left(Z\right|\theta ,\alpha ,\delta ,\tau ,\omega )=\prod_{j}^{J}\prod_{i}^{I}P\left[Z_{ij}=z\right|\theta_{j}]$$

where $P\left[Z_{i}=z\right|\theta_{j}]$ is the MGGUM probability, Z is the response data, α,δ,τ are the item parameters, θ are the person parameters, and ω is the variance–covariance of the prior distribution of θ. For model identification, the mean and variance of person parameters on each dimension were fixed to zero and one, respectively. Correlations between dimensions were freely estimated.

### 1.3. Incorporation of Covariates

To improve MGGUM estimation accuracy and obtain accurate estimates of the relationship between traits and covariates (e.g., gender, age, educational levels), incorporating covariates into the estimation process can be a promising approach. In both the dominance IRT and the unidimensional unfolding IRT frameworks, studies have found that incorporating covariates into the estimation process improves the accuracy of trait score estimates (Joo et al. 2022; Usami 2011), especially when covariates and traits were moderately to highly correlated (Curran et al. 2016). Similar to the idea of borrowing information from correlated traits to improve estimation, the incorporation of covariates could improve estimation by borrowing information from correlated covariates. We expect the same to be true for the MGGUM. The incorporation of covariates can also facilitate the examination of the relationship between traits and covariates. Instead of estimating trait scores and then correlating the estimated trait scores with covariates, which may result in downward bias, incorporating covariates into the estimation process can account for the measurement error of the traits, leading to more accurate estimates of the relationships between traits and covariates. However, it is unknown how accurately the relationship between covariates and traits can be estimated and how much improvement in MGGUM trait score estimation can be gained by incorporating covariates. Therefore, this research examines the accuracy of the Bayesian algorithm in capturing the effects of covariates and assesses their impact on MGGUM estimation. In the package *bmggum*, the relationship between traits and covariates can be formulated as

(4) $$\theta_{jd}=\sum_{1}^{p}\beta_{pd}X_{jp}+{ℇ}_{jd}$$

where θ_jd_ is the latent trait level of person j on the d^th^ dimension, $X_{jp}$ is the p^th^ covariate of person j, $\beta_{pd}$ is the regression coefficient of the relationship between the d^th^ dimension and the p^th^ covariates, p is the number of covariates, ${ℇ}_{jd}$ is the residual and is assumed to be normally distributed with a mean of 0 and variance of $\sigma^{2}$.

### 1.4. Bayesian Model Fit Diagnostics

Examining model fit and selecting the best fitting model is crucial for accurate parameter estimation (Nye et al. 2020). For example, studies have shown that fitting a unidimensional GGUM to multidimensional data could lead to estimation bias (Carter and Zickar 2011; Joo et al. 2022). However, if dimensions are highly correlated, fitting a unidimensional GGUM to the data may have little effect on estimation accuracy, as a high correlation indicates that the dimensions largely reflect the same latent trait. In this research, two Bayesian fit indices, namely the widely available information criterion (WAIC; Watanabe and Opper 2010) and the leave-one-out cross-validation (LOO; Geisser and Eddy 1979) that are included in the R package *bmggum* are examined to assist with model selection.

WAIC and LOO are two Bayesian model selection indices that involve posterior distributions and are considered more appropriate for Bayesian estimation, which involves prior distributions, compared to frequentist indices such as Akaike’s information criterion (AIC; Akaike 1973) and Bayesian information criterion (BIC; Schwarz 1978), which are computed based on maximum likelihood estimation results (Nye et al. 2020; Spiegelhalter et al. 2003). In contrast to the deviance information criterion (DIC; Spiegelhalter et al. 2003), which is computed using a point estimate from the posterior distribution, WAIC and LOO use the entire posterior distribution and are therefore theoretically preferred over DIC (Luo and Al-Harbi 2017). Studies comparing these model selection indices have found that WAIC and LOO perform reasonably well and better than the likelihood ratio test (LRT), AIC, and BIC for dichotomous dominance IRT model selection (e.g., Luo and Al-Harbi 2017; Yong 2018). However, their performance has yet to be evaluated for the GGUM. Their performance in selecting the best fitting polytomous unfolding model (i.e., MGGUM) is therefore evaluated in this research.

## 2. Study 1. Model Estimation Accuracy

Study 1 examined the estimation accuracy of the Bayesian algorithm implemented in *bmggum* and the effect of multidimensional estimation and covariate incorporation on MGGUM parameter estimation under realistic conditions.

### 2.1. Method

*Study design*. Seven factors were manipulated to produce 192 conditions: (1) sample size (200, 500, 1000), (2) the number of traits (2, 5), (3) the number of items per trait (5, 10), (4) the number of response options (2, 4), (5) missing data as the proportion of cells missing (0, 0.20), (6) the correlations between traits (0, 0.50), and (7) the correlations between traits and covariates (0, 0.25). The number of covariates was fixed at 2. The sample sizes were chosen based on previous research on GGUM parameter estimation (de la Torre et al. 2006; Joo et al. 2017; Roberts et al. 2000; Roberts and Thompson 2011; Stark et al. 2005). A sample size of 200 is considered small for the GGUM, whereas 500 is slightly larger than the minimum requirement of 400, and 1000 is considered reasonably large. Two and five dimensions were representative of the dimensionality of key constructs. For example, organizational citizenship behavior (OCB) consists of two dimensions, and personality is known to have five broad dimensions. Five items per trait is typical of short scales such as the short version of the Organizational Citizenship Behavior Scale (Spector et al. 2010), and ten items per trait is also common for personality scales like the IPIP-50 (Ehrhart et al. 2008). The number of response options was selected following previous research (e.g., de la Torre et al. 2006; Joo et al. 2017). To reflect situations with no missing data and with a reasonable amount of missing data, 0% and 20% of missing data were simulated, respectively. The correlation of 0.50 between traits was selected to reflect a moderate degree of correlation between latent factors, and the correlation of 0.25 between traits and covariates was selected to reflect a realistic degree of correlation between focal factors and external variables. The conditions in which the correlations between traits are 0 are equivalent to estimating each trait separately. The conditions in which the correlations between traits and covariates are 0 are equivalent to having no covariates.

*Data generation*. In line with previous simulation studies (e.g., Cao et al. 2018; Joo et al. 2017; Nye et al. 2020; Roberts et al. 2002; Tay et al. 2011), item discrimination parameters (α) were sampled from uniform distributions *U*(0.50, 2.00). Item location parameters (δ) were sampled from *U*(−2.00, 2.00). In the conditions with two response options, threshold parameters (τ) were randomly drawn from *U*(−3.00, −1.00). In the conditions with four response options, threshold parameters were randomly drawn from *U*(−3.50, −2.50), *U*(−2.50, −1.50), and *U*(−1.50, −0.50), respectively. Person parameters (θ) and covariates were sampled from a multivariate normal distribution with means and variances fixed to 0 and 1, respectively. The correlation between covariates was set to 0, the correlations between traits were set to 0 or 0.50, and the correlations between traits and covariates were set to 0 or 0.25 depending on the conditions. Generated item and person parameters were then used to generate response data following Equation (1).

*Estimation.* The *bmggum* package allows for the specification of item directions and priors to facilitate model convergence. In this study, items were classified as negative if their location parameters (δ) were lower than −1.5, positive if their δs were higher than 1.5, and neutral if their δs fell between −1.5 and 1.5. For prior distributions, we examined prior choices in previous studies and fine-tuned them through trial and error to ensure that they consistently resulted in model convergence in this study. Specifically, *N*(−1, 1) and *N*(1, 1) were used as the prior distributions for the location parameters of negative and positive items, respectively. In addition, a lower bound of 0 was imposed on the location parameters of positive items, and an upper bound of 0 was imposed on the location parameters of negative items. For neutral items, an *N*(0, 1) prior distribution was used with no bounds imposed. For threshold parameters, in the 2-response-options conditions, τ *~ N*(−2, 2), and in the 4 -response-options conditions, $\tau_{1}$ *~ N*(−3, 2), $\tau_{2}$ *~ N*(−2, 2), and $\tau_{3}$ *~ N*(−1, 2). A log*N*(0, 0.5) prior distribution was used for discrimination parameters. For person parameters, **θ** ~ *MVN*(β**X**, ω), β ~ *MVN*(0, 1), ω ~ lkj_corr_cholesky(1). Random initial values generated by *bmggum* were used. Based on the preliminary trials, 2000 iterations with 2 chains were sufficient to achieve convergence. Therefore, in this study, 2000 iterations with 2 chains were performed and the first 1000 iterations were discarded as burn-in. Model convergence was assessed using the Gelman–Rubin diagnostic index (Gelman and Rubin 1992), which compares the variability of samples after burn-in within parallel chains with the variability between parallel chains. If the ratio of variability between parallel chains to variability within parallel chains is less than 1.05, we considered it as evidence for model convergence. If a certain replication failed to converge, it was discarded, and an additional replication was conducted until 100 valid replications were obtained per condition. Overall, model nonconvergence was rare, specifically less than 4%, in this study.

*Analysis.* To evaluate the estimation accuracy of parameters in each condition, three indices were computed for each replication: Pearson correlation (Cor) between true and estimated parameters, bias, and absolute error (Ae). Bias is defined as the average difference between true and estimated parameters, and Ae is the average absolute difference between true and estimated parameters. For example, bias ($\hat{\alpha }$) $=\frac{\sum_{j}\left(\hat{\alpha }-\alpha \right)}{S}$, Ae ($\hat{\alpha }$) $=\frac{\sum_{j}\left|\hat{\alpha }-\alpha \right|}{S}$, where S is the total number of items, j represents the j^th^ item, $\hat{\alpha }$ is the parameter estimate, and α is the true parameter. To have a single value of each estimation accuracy index for each condition, the obtained Cor, bias, and Ae values for item and person parameter estimates were averaged across replications and dimensions. Larger Cor and smaller bias and Ae indicate more accurate parameter estimation. The power/Type I error rates for detecting the correlations between traits and covariates were also computed by examining whether the 95% confidence interval of the posterior distribution of the regression coefficients included zero. If zero was included in the 95% confidence interval, it was considered statistically non-significant; if zero was not included in the 95% confidence interval, it was considered statistically significant.

### 2.2. Results

*Person parameter.* Table 1 presents the results for person parameter θs. The correlations (Cors) between true and estimated θs ranged from 0.52 to 0.93 and absolute errors (Aes) ranged from 0.28 to 0.66 across conditions. Biases were all 0 due to the counterbalance of positive biases and negative biases. As expected, person parameter estimation improved when correlated traits were estimated simultaneously. The beneficial effects of simultaneous estimation were particularly salient when the number of items per trait and the number of response options were small, or when there were missing data. For example, person parameters were estimated least accurately in the condition with five items per trait, two response options, 20% missing data, no correlation between traits, and zero-effect covariates, resulting in Cors = 0.52, bias = 0, and Aes = 0.66. In contrast, when the correlations between traits were changed from 0.00 to 0.50, indicating the correlated traits were estimated simultaneously, Cors increased from 0.52 to 0.63, Aes decreased from 0.66 to 0.61, and bias was 0. This confirmed the positive effect of multidimensional estimation on person parameter estimation. In conditions where the number of items per trait and the number of response options were high (e.g., 10 items and four response options), the improvement in person parameter estimation provided by multidimensional estimation was less obvious. The incorporation of covariates also improved person parameter estimation. When the correlations between trait and covariates were changed from 0.00 to 0.25, Cors increased from 0.52 to 0.60, Aes decreased from 0.66 to 0.63, and bias was still 0. It is worth noting that this improvement was achieved using only two weak covariates (i.e., 0.25 correlation with the traits). With more or stronger covariates, more substantial improvement can be expected. When the traits were correlated, person parameter estimation also improved with an increasing number of traits. This is because the inclusion of more correlated traits provides more trait-relevant information to improve person parameter estimation. As expected, more items per trait, more response options, and fewer missing data were associated with better person parameter estimation. The results were also depicted in Figure A1 of Appendix C in the Supplementary Material.

It is well-known that person parameters at the two extreme ends of the latent trait continuum are harder to estimate than person parameters in the middle (e.g., Joo et al. 2022). To evaluate the estimation of person parameters more clearly, we divided the overall θs into extreme θs (i.e., θs > 1.282 or <−1.282, which correspond to the top 10% and the bottom 10%, respectively) and middle θs (i.e., −1.282 ≤ θs ≤ 1.282). The results are included in Table A1 of Appendix B in the Supplementary Material. A similar pattern of results as overall θs was observed for middle θs. However, worse estimation was observed for both negative and positive extreme θs, with Cors ranging from 0.00 to 0.67 and 0.00 to 0.66, Aes ranging from 0.31 to 1.34 and 0.31 to 1.32, and biases ranging from 0.03 to 1.34 and −1.32 to −0.04, respectively. The biases indicate that negative extreme θs tended to be estimated more positively, while positive extreme θs tended to be estimated more negatively. It is worth noting that the low correlations for extreme θs were a joint product of estimation error and range restriction, which is common for most IRT models. Because of the inadequate estimation accuracy of extreme θs, the effects of multidimensional estimation, incorporating covariates, and increasing the number of correlated traits on extreme θ estimation were more salient. For example, when the traits were estimated simultaneously, Cors increased by as much as 0.24, from 0.11 to 0.35, Aes decreased by 0.16 from 0.75 to 0.59, and biases decreased by 0.19 from 0.72 to 0.53 compared to estimating each trait separately. Similarly, when covariates were incorporated, Cors increased by as much as 0.17, from 0.01 to 0.18, Aes decreased by 0.18 from 1.16 to 0.98, and biases decreased by 0.19 from −1.16 to −0.97. Extreme θs were also estimated more accurately with five traits than with two traits when the traits were correlated and estimated simultaneously. The results for person parameter recovery were also visualized in Figures A2–A9 of Appendix C in the Supplemental Material. Note that these results may not be considered satisfactory due to the intentionally selected challenging conditions in this study. The main purpose was to demonstrate the beneficial effects of simultaneous multidimensional estimation and the incorporation of covariates. When making decisions about individuals based on these trait scores, it is recommended that researchers use longer tests and more refined response options aside from simultaneous estimation of the MGGUM and the incorporation of informative covariates.

*Item parameters.* Due to space limitations, the results for item parameters α, δ, and τ are included in Tables A2–A4 of Appendix B in the Supplemental Material. The correlations between true and estimated αs ranged from 0.42 to 0.96, with Aes ranging from 0.07 to 0.28 and biases ranging from −0.16 to 0. The correlations between true and estimated δs ranged from 0.95 to 1, with Aes ranging from 0.11 to 0.35 and biases ranging from −0.02 to 0.02. The correlations between true and estimated τs ranged from 0.76 to 0.93, with Aes ranging from 0.14 to 0.36 and biases ranging from −0.29 to −0.04. Similar to person parameter estimation, multidimensional estimation, incorporating covariates, and increasing the number of correlated traits led to higher correlations between true and estimated αs, especially in conditions where αs were not well estimated (i.e., Cors below 0.80). However, the effects did not extend to the biases and Aes of αs, suggesting that multidimensional estimation, incorporating covariates, and increasing the number of correlated traits enhanced the accuracy of the rank order of αs, but not the exact estimates themselves. The effects did not extend to the estimation of δs and τs. This might be attributed to the high correlation between true and estimated δs and τs, leaving little room for improvement. As expected, a larger sample size, more response options, more items per trait, and fewer missing data were associated with more accurate estimation of αs and δs. Similarly, the estimation of τs improved with larger sample size, more items per trait, and fewer missing data. However, the number of response options had a negative effect on the estimation of τs when the sample size was small (i.e., 200, 500) and a positive effect when the sample size was large (i.e., 1000). This might be because, as the number of response options increases, so does the number of τs to be estimated, thus requiring a larger sample size for adequate parameter estimation accuracy. The results for item parameter recovery were plotted in Figures A10–A12 of Appendix C in the Supplemental Material. We also conducted separate analyses for items with negative and positive location parameters, and we observed the same pattern as the overall item parameters, except for τs of negative items. In the case of negative items, the number of response options had a negative effect on the estimation of τs, regardless of the sample size. The corresponding results can be found in Tables A8–A13 of Appendix B in the Supplemental Material.

*Correlations between traits and correlations between traits and covariates.* The results for the correlations between traits and the correlations between traits and covariates were included in Tables A5 and A6 of Appendix B in the Supplemental Material. The biases ranged from −0.08 to 0.04 and −0.01 to 0.01, and Aes ranged from 0.01 to 0.20 and 0.01 to 0.11 for the correlations between traits and the correlations between traits and covariates, respectively, indicating that the correlations between traits and the correlations between traits and covariates can be estimated with high accuracy, even in the conditions with small sample sizes, small numbers of items per trait and response options, and missing data. As expected, the estimation of both types of correlations improved with more response options, larger sample size, more items per trait, and fewer missing data. Figures A13 and A14 of Appendix C in the Supplemental Material provide a visualization of the results.

*Power/Type I error rates.* The power/Type I error rates for detecting the correlations between traits and covariates are presented in Table A7 of Appendix B in the Supplemental Material. When the correlations between traits and covariates were 0.25, the power to detect the relationships was mostly high, ranging from 0.89 to 1, except for the conditions with 200 respondents, five items per trait, and two response options, where the power ranged from 0.48 to 0.67. As expected, larger sample sizes, more response options, more items per factor, and fewer missing data resulted in higher power. While we had good statistical power to detect the effects of covariates, the Type I error rates were underestimated in most conditions. Specifically, all of them were close to zero instead of 5% when the true effects of covariates on the focal factor were zero.

## 3. Study 2. Model Selection Accuracy

Study 2 investigated the effectiveness of the two Bayesian model selection indices, WAIC and LOO, in selecting the GGUM with appropriate dimensionality.

### 3.1. Method

*Study design*. Five factors were manipulated to produce 96 conditions: (1) sample size (200, 500, 1000), (2) the number of items per trait (5, 10), (3) the number of response options (2, 4), (4) missing data as the proportion of cells missing (0, 0.20), and (5) the correlations between traits (0.30, 0.60, 0.90, 1). The number of traits was fixed at 2.

*Data generation.* The same data generation process as in Study 1 was used, with the number of traits fixed at 2 to generate data for two dimensions.

*Estimation.* The generated data were fitted to both a unidimensional and a 2-dimensional GGUM using the same estimation process employed in Study 1.

*Analysis.* The model fit information for WAIC and LOO was extracted from both the unidimensional and 2-dimensional GGUMs that were fitted to the generated data. Smaller WAIC and LOO values for the 2-dimensional GGUM indicated that the 2-dimensional GGUM had a better fit, thus the true model was identified, except for when the correlation between traits was 1. A correlation of 1 indicated that the traits were identical, and therefore the unidimensional and 2-dimensional GGUMs would fit the data equally well. As WAIC and LOO do not penalize model complexity, it was expected that the unidimensional and 2-dimensional GGUMs would produce similar model fits when the correlation between traits was 1, resulting in a power of around 0.50. The power of WAIC and LOO in identifying the correct model (i.e., 2-dimensional GGUM) was computed by calculating the percentage of times that WAIC and LOO correctly selected the model across 100 replications for each condition.

### 3.2. Results

Table 2 presents the power of WAIC and LOO in correctly identifying the true model. When the correlation between the two traits was 0.30, the power of WAIC for identifying the correct model (i.e., 2-dimensional GGUM) ranged from 0.97 to 1, and the power of LOO ranged from 0.94 to 1. When the correlation was 0.60, the power of WAIC ranged from 0.89 to 1, and the power of LOO ranged from 0.76 to 1. When the correlation was 0.90, the power of WAIC and LOO ranged from 0.62 to 1 and 0.39 to 1, respectively. When the two traits were perfectly correlated, the power of WAIC ranged from 0.28 to 0.63, and the power of LOO ranged from 0.45 to 0.77. It is evident that both indices displayed high power in identifying the true model, with WAIC showing relatively higher power than LOO across conditions. As expected, the power of both indices decreased as the correlations between traits increased, especially when the sample size, the number of items per trait, and the number of response options were small. This is because, as the correlations between traits increases, it becomes more difficult to differentiate between them, resulting in a smaller difference between fitting a unidimensional GGUM and a 2-dimensional GGUM. When the correlation was 1, both the unidimensional GGUM and the 2-dimensional GGUM were considered correct, resulting in low power of WAIC and LOO, which were computed using the 2-dimensional GGUM as the correct model.

## 4. Study 3. Empirical Illustration

To further illustrate the utility of the Bayesian algorithm implemented in *bmggum*, we analyzed a multidimensional personality dataset using *bmggum*. Covariates were included in the dataset to estimate the relationship between the traits and covariates. The results were further evaluated by comparing them with those obtained using *GGUM2004*, *GGUM*, and *mirt*.

### 4.1. Method

*Data.* The data were obtained from Cao et al. (2015), which included 355 undergraduate respondents from a university located in the Midwest region of the United States. The age of the respondents ranged from 18 to 28, with a mean age of 19.35. Of the respondents, 71% were females. In terms of racial and ethnic backgrounds, 70.1% identified as White, 15.2% as Asian, 5.9% as Hispanic or Latino, 5.6% as Black or African American, and 3.1% as belonging to other races or ethnicities.

*Measures.* Eleven items were selected from each of the two personality facets (i.e., the orderliness facet of conscientiousness and the assertiveness facet of extraversion) based on their good performance in Cao et al. (2015). For each facet, 4 items were positively worded (i.e., δ > 1.5), 4 items were negatively worded (i.e., δ < −1.5), and 3 items were intermediate. Each item was rated on a 4-point Likert scale ranging from 1 (*Strongly disagree*) to 4 (*Strongly agree*).

*Analysis.* Due to the small number of respondents endorsing certain response options, we dichotomized the responses by coding *strongly disagree* and *disagree* as 0, and *strongly agree* and *agree* as 1, following Cao et al. (2015). An MGGUM with 2 dimensions was fitted to the data using *bmggum*. Two covariates, age and gender, were also included in the dataset. As the package currently can only handle missing data in response data and not in covariates, two respondents with missing covariate data were deleted. An annotated step-by-step R script can be found in Appendix A of the Supplemental Material. We also fitted a unidimensional GGUM to each facet using *GGUM*, *GGUM2004*, and *mirt* and recorded the corresponding item parameters and person scores.

### 4.2. Results

*Item parameters.* Table 3 presents the estimated item parameters and their standard errors obtained using *bmggum*, *GGUM2004*, *GGUM*, and *mirt*. As can be seen in Table 3, there were items exhibiting low, medium, and high levels of discrimination. In the results obtained using *GGUM2004* and *GGUM*, extreme item parameter estimates and huge standard errors were observed, which have been highlighted in bold. These abnormal estimates were primarily observed for items located at the two extreme ends of the latent trait continuum (i.e., items with locations greater than 2 or less than −2). In comparison, the estimates and standard errors obtained using *bmggum* and *mirt* were less extreme.

*Person parameter.* The person parameter estimates obtained using *bmggum* (with and without covariates), *GGUM2004*, *GGUM,* and *mirt* showed high correlations ranging from 0.987 to 0.999.

*Correlations between traits and correlations between traits and covariates.* The *bmggum* results showed a moderate correlation between orderliness and assertiveness, with r = 0.20 and SE = 0.06 when incorporating gender and age as covariates, and r = 0.18 and SE = 0.06 without covariates. Gender differences were observed for both orderliness and assertiveness, with women being more orderly than men (r = 0.45, SE = 0.13), and men being more assertive than women (r = −0.24, SE = 0.13). Age differences in orderliness (r = −0.01, SE = 0.02) and assertiveness (r = 0.04, SE = 0.02) were negligible. We also estimated the trait scores using *GGUM2004*, *GGUM*, and *mirt* and then computed the same correlations. The results showed that orderliness and assertiveness were correlated with r = 0.13, 0.14, and 0.18, respectively. A significant but smaller gender difference was observed for orderliness, with women being more orderly than men (r*_GGUM2004_* = 0.34, SE*_GGUM2004_* = 0.10; r*_GGUM_* = 0.34, SE*_GGUM_* = 0.10; r*_mirt_* = 0.32, SE*_mirt_* = 0.10), and no significant gender difference was found for assertiveness (r*_GGUM2004_* = −0.18, SE*_GGUM2004_* = 0.10; r*_GGUM_* = −0.18, SE*_GGUM_* = 0.10; r*_mirt_* = −0.14, SE*_mirt_* = 0.11). Age differences in orderliness (r*_GGUM2004_* = 0.01, SE*_GGUM2004_* = 0.04; r*_GGUM_* = 0.01, SE*_GGUM_* = 0.04; r*_mirt_* = 0.01, SE*_mirt_* = 0.04) and assertiveness (r*_GGUM2004_* = 0.03, SE*_GGUM2004_* = 0.04; r*_GGUM_* = 0.03, SE*_GGUM_* = 0.04; r*_mirt_* = 0.04, SE*_mirt_* = 0.04) were not significant.

In sum, estimating the MGGUM with covariates using *bmggum* was found to effectively address the issue of extreme item parameter estimates and huge standard errors observed in *GGUM2004* and *GGUM.* Stronger correlations between traits and covariates, as well as between traits themselves, were also observed with *bmggum*, despite highly correlated person parameter estimates obtained from different software programs. The performance of *bmggum* and *mirt* were comparable, though further research is needed to evaluate *mirt* in high dimensional data.

## 5. Discussion

Given the abundance of multidimensional noncognitive constructs in the literature and the difficulty of estimating the MGGUM, this research evaluated a Bayesian algorithm implemented in the R package *bmggum* that can estimate the MGGUM with covariates. Simulation studies showed that the MGGUM parameters can be accurately estimated using the algorithm. It was also found that WAIC and LOO were effective in selecting the best-fitting model. The empirical example also demonstrated the utility of the new algorithm, and a step-by-step tutorial on how to fit the MGGUM with covariates is included in Appendix A of the Supplemental Material to make this approach more accessible. Overall, this research made an important contribution towards the measurement of noncognitive constructs.

### 5.1. The Benefit of Multidimensional Estimation and the Incorporation of Covariates

Consistent with de la Torre et al. (2006), this research showed that Bayesian estimation was effective at eliminating extreme estimates of item parameters and standard errors, which is one of the major limitations of existing estimation approaches. Central to this research, we found that the MGGUM item and person parameters, correlations between traits, and relationships between traits and covariates can be well estimated using the Bayesian algorithm implemented in *bmggum*. Moreover, estimating multiple dimensions simultaneously and incorporating relevant covariates into the estimation process improved estimation accuracy, which is consistent with the findings in previous IRT literature (e.g., Curran et al. 2016; Wang et al. 2004). This approach proved especially useful when the number of items measuring each trait or the number of response options per item were small, or when there were missing data, which are common in psychological research. Among all the parameters, person parameter estimates benefited the most. Particularly, it was highly advantageous for estimating person parameters at the two extremes of the latent trait continuum, which are critical for academic and personnel selection but notoriously difficult to estimate.

Multidimensional estimation and the incorporation of covariates improve estimation because they provide additional information about the focal traits. For example, when estimating Assertiveness trait scores using the unidimensional GGUM without covariates, only items measuring Assertiveness are involved in the estimation process, which may lead to less accurate estimates when there are limited items and response options. However, when estimating Assertiveness trait scores using the MGGUM with covariates, items measuring other correlated traits (e.g., Sociability) and covariates can provide information about the Assertiveness trait in addition to the focal Assertiveness items, which is especially useful when the information provided by the focal items is limited.

It was also found that correlations between traits and between traits and covariates can be estimated accurately using the Bayesian algorithm, even in the least favorable conditions. This advantage is analogous to the benefit of using Structural Equation Modeling (SEM) over observed-score-based path models due to the measurement error control. Therefore, researchers who are primarily interested in structural parameters (e.g., relationships between traits) than measurement parameters (e.g., item parameters) or individual scores are recommended to use this algorithm.

### 5.2. Model Selection

This research also investigated the effectiveness of the two Bayesian model selection indices, namely WAIC and LOO, in identifying the most appropriate model. The results showed that both indices demonstrated high power in identifying the best-fitting model, making them useful in selecting models as well as exploring the dimensionality of unfolding data. By fitting the MGGUM with different numbers of dimensions to the data, the model that fits the data the best can be identified by comparing the WAIC and LOO values. However, as these two indices do not include a penalty for model complexity, they are not effective in selecting between equivalent models. In such cases, researchers may opt for the more parsimonious model.

### 5.3. Implications

This research has important implications for both research and practice. In empirical research, the examination of relationships between traits or between traits and covariates is common for testing and refining theories, making the more accurate estimates of these correlations obtained from MGGUM with covariates particularly valuable for facilitating theory development. When the traits are noncognitive (e.g., personality, motivation, person-organization fit, vocational interests), the *bmggum* package can be used to obtain more accurate estimates of the relationships. In practice, ranking individuals accurately, especially at the top and bottom ends, is essential for selection decisions. Therefore, MGGUM estimation can be used for estimating data on multidimensional noncognitive traits to obtain more accurate person parameters, especially person parameters at the two extremes of the latent trait continuum, facilitating fair and optimal decision-making. If data on additional variables are available, MGGUM estimation with covariates can further improve scoring accuracy.

### 5.4. Limitations and Future Directions

Despite the strengths of this research, there are several limitations worth noting. First, the effect of the number of correlated covariates on MGGUM estimation accuracy was not examined in this research. It is expected that the larger the number of correlated covariates, the higher the parameter estimation accuracy, simply because the more information, the better. Second, this research only focused on the advantage of between-item multidimensionality. However, within-item multidimensionality, where an item can measure more than one trait, is also common in noncognitive measures (Zhang et al. 2023). Future research is encouraged to expand the MGGUM to handle within-item multidimensionality.

### 5.5. Conclusions

The current study contributes to the literature by providing evidence for the estimation accuracy of the R package *bmggum* and demonstrating the advantages of multidimensional estimation and the incorporation of covariates. It is hoped that this research will facilitate future exploration of (M)GGUM applications in noncognitive assessment.

## Figures and Tables

**Table 1:** Estimation accuracy for person parameters.

Missing	r	Traits	Beta	Cor				Bias				Ae			
				Items = 5		Items = 10		Items = 5		Items = 10		Items = 5		Items = 10	
				RO = 2	RO = 4	RO = 2	RO = 4	RO = 2	RO = 4	RO = 2	RO = 4	RO = 2	RO = 4	RO = 2	RO = 4
0	0	2	0.00	0.60	0.84	0.75	0.92	0.00	0.00	0.00	0.00	0.62	0.41	0.50	0.29
			0.25	0.65	0.85	0.78	0.93	0.00	0.00	0.00	0.00	0.59	0.40	0.48	0.30
		5	0.00	0.59	0.84	0.75	0.92	0.00	0.00	0.00	0.00	0.62	0.41	0.50	0.29
			0.25	0.65	0.86	0.78	0.93	0.00	0.00	0.00	0.00	0.59	0.40	0.48	0.29
	0.5	2	0.00	0.64	0.86	0.78	0.93	0.00	0.00	0.00	0.00	0.59	0.39	0.48	0.29
			0.25	0.67	0.86	0.79	0.93	0.00	0.00	0.00	0.00	0.58	0.39	0.47	0.29
		5	0.00	0.69	0.87	0.81	0.93	0.00	0.00	0.00	0.00	0.56	0.38	0.46	0.28
			0.25	0.70	0.87	0.81	0.93	0.00	0.00	0.00	0.00	0.56	0.38	0.46	0.29
0.2	0	2	0.00	0.54	0.79	0.69	0.90	0.00	0.00	0.00	0.00	0.65	0.46	0.55	0.33
			0.25	0.61	0.81	0.73	0.90	0.00	0.00	0.00	0.00	0.62	0.45	0.52	0.33
		5	0.00	0.52	0.78	0.69	0.89	0.00	0.00	0.00	0.00	0.66	0.46	0.55	0.33
			0.25	0.60	0.81	0.73	0.91	0.00	0.00	0.00	0.00	0.63	0.45	0.52	0.33
	0.5	2	0.00	0.58	0.81	0.73	0.91	0.00	0.00	0.00	0.00	0.63	0.44	0.52	0.32
			0.25	0.63	0.82	0.75	0.91	0.00	0.00	0.00	0.00	0.61	0.44	0.51	0.33
		5	0.00	0.63	0.83	0.77	0.91	0.00	0.00	0.00	0.00	0.60	0.42	0.50	0.32
			0.25	0.66	0.83	0.77	0.91	0.00	0.00	0.00	0.00	0.59	0.43	0.49	0.32

*Note.* Missing = The proportion of missing data; r = Correlation between traits; Traits = No. of traits; Beta = Regression coefficients; Items = No. of items; RO = No. of response options; Cor = Correlation between true person parameters and estimated person parameters; Bias = Average difference between true and estimated parameters; Ae = average absolute difference between true and estimated parameters.

**Table 2:** Power/Type I Error Rate to identify the correct model.

Sample Size	Missing	r	WAIC				LOO			
			Items = 5		Items = 10		Items = 5		Items = 10	
			RO = 2	RO = 4	RO = 2	RO = 4	RO = 2	RO = 4	RO = 2	RO = 4
200	0.00	0.30	1.00	1.00	1.00	1.00	1.00	1.00	1.00	1.00
		0.60	0.98	0.99	1.00	1.00	0.88	0.97	1.00	1.00
		0.90	0.62	0.96	0.77	1.00	0.39	0.92	0.64	1.00
		1.00	0.54	0.53	0.63	0.58	0.66	0.71	0.73	0.68
	0.20	0.30	0.97	1.00	1.00	1.00	0.94	1.00	1.00	1.00
		0.60	0.89	1.00	0.99	1.00	0.76	1.00	0.99	0.99
		0.90	0.71	0.95	0.71	1.00	0.49	0.83	0.63	0.98
		1.00	0.55	0.36	0.59	0.61	0.75	0.61	0.72	0.73
500	0.00	0.30	1.00	1.00	1.00	1.00	0.99	1.00	1.00	1.00
		0.60	0.94	1.00	0.98	1.00	0.92	0.99	0.98	1.00
		0.90	0.85	0.99	0.86	1.00	0.59	0.97	0.76	1.00
		1.00	0.56	0.33	0.62	0.59	0.77	0.55	0.70	0.72
	0.20	0.30	1.00	1.00	1.00	1.00	1.00	1.00	1.00	1.00
		0.60	0.99	1.00	0.99	1.00	0.95	1.00	0.99	1.00
		0.90	0.82	0.97	0.90	1.00	0.50	0.92	0.82	1.00
		1.00	0.29	0.33	0.55	0.50	0.67	0.62	0.71	0.57
1000	0.00	0.30	1.00	1.00	1.00	1.00	1.00	1.00	1.00	1.00
		0.60	0.97	0.99	1.00	1.00	0.96	0.99	1.00	1.00
		0.90	0.89	1.00	0.97	1.00	0.68	1.00	0.95	1.00
		1.00	0.49	0.34	0.55	0.51	0.71	0.58	0.59	0.64
	0.20	0.30	1.00	1.00	1.00	1.00	0.99	1.00	1.00	1.00
		0.60	0.99	1.00	1.00	1.00	0.98	1.00	1.00	1.00
		0.90	0.93	0.98	0.95	1.00	0.69	0.95	0.85	1.00
		1.00	0.32	0.28	0.56	0.37	0.63	0.55	0.70	0.45

*Note.* Missing = The proportion of missing data; r = Correlation between traits; Items = No. of items; RO = No. of response options.

**Table 3:** Estimated item parameters and standard errors (SE) using the four software programs.

Items	Alpha				Delta				Tau			
	bmggum	ggum 2004	ggum	mirt	bmggum	ggum 2004	ggum	mirt	bmggum	ggum 2004	ggum	mirt
Order1	1.57 (0.25)	1.95 (0.37)	1.96 (0.37)	1.79 (0.30)	1.96 (0.48)	1.53 (0.30)	1.52 (0.29)	1.45 (0.25)	−1.98 (0.49)	−1.57 (0.29)	−1.56 (0.29)	1.47 (0.24)
Order2	1.10 (0.17)	1.23 (0.67)	1.23 (0.67)	1.15 (0.19)	2.15 (0.49)	**2.69 (17.50)**	**2.59 (15.90)**	1.71 (0.35)	−3.18 (0.53)	**−3.67 (17.77)**	**−3.57 (16.18)**	2.67 (0.40)
Order3	1.49 (0.23)	1.56 (0.37)	1.57 (0.50)	1.55 (0.26)	2.11 (0.50)	**2.37 (6.27)**	**2.58 (10.65)**	1.53 (0.35)	−2.29 (0.51)	**−2.57 (6.44)**	**−2.78 (10.89)**	1.69 (0.35)
Order4	1.10 (0.20)	1.23 (0.23)	1.22 (0.23)	1.24 (0.24)	1.58 (0.45)	1.24 (0.25)	1.24 (0.25)	1.16 (0.23)	−1.21 (0.42)	−0.94 (0.20)	−0.94 (0.20)	0.86 (0.18)
Order5	1.26 (0.22)	1.24 (0.54)	1.24 (0.56)	1.41 (0.40)	−3.21 (0.49)	**−4.53 (22.29)**	**−4.42 (20.45)**	−1.60 (0.34)	−0.46 (0.41)	**−1.84 (23.33)**	**−1.72 (21.55)**	−0.84 (0.44)
Order6	1.11 (0.18)	1.25 (0.66)	1.26 (0.63)	1.15 (0.21)	−2.48 (0.54)	**−3.53 (14.42)**	**−3.72 (16.77)**	−1.91 (0.41)	−1.18 (0.58)	**−2.41 (15.22)**	**−2.62 (17.49)**	0.65 (0.39)
Order7	1.94 (0.32)	2.61 (0.60)	2.61 (0.57)	2.25 (0.39)	−2.54 (0.44)	**−2.74 (9.90)**	**−2.68 (7.82)**	−2.06 (0.28)	−1.43 (0.46)	**−1.79 (10.03)**	**−1.74 (7.95)**	1.05 (0.28)
Order8	1.96 (0.33)	2.71 (0.55)	2.71 (0.55)	2.34 (0.44)	−2.29 (0.45)	−1.82 (0.27)	−1.82 (0.27)	−1.79 (0.25)	−1.19 (0.45)	−0.90 (0.24)	−0.89 (0.24)	0.80 (0.21)
Order9	0.68 (0.14)	0.55 (0.19)	0.55 (0.19)	0.63 (0.16)	−0.74 (0.33)	−0.82 (0.46)	−0.82 (0.45)	−0.68 (0.30)	−0.81 (0.25)	−0.73 (0.28)	−0.73 (0.28)	0.70 (0.22)
Order10	1.69 (0.33)	2.45 (0.47)	2.45 (0.48)	2.21 (0.45)	−0.01 (0.10)	−0.03 (0.07)	−0.03 (0.07)	−0.03 (0.09)	−1.29 (0.10)	−1.15 (0.07)	−1.15 (0.07)	1.18 (0.08)
Order11	1.57 (0.29)	2.20 (0.43)	2.20 (0.43)	1.91 (0.37)	0.26 (0.12)	0.22 (0.08)	0.22 (0.08)	0.21 (0.10)	−1.23 (0.11)	−1.11 (0.08)	−1.10 (0.08)	1.13 (0.09)
Assertiveness1	2.04 (0.29)	2.81 (0.44)	2.82 (0.45)	2.46 (0.37)	1.70 (0.48)	1.29 (0.26)	1.28 (0.26)	1.20 (0.22)	−2.51 (0.49)	−2.01 (0.27)	−2.00 (0.27)	1.93 (0.23)
Assertiveness2	1.21 (0.22)	1.56 (0.35)	1.31 (0.40)	1.46 (0.31)	1.06 (0.44)	0.84 (0.34)	**3.58 (26.53)**	.63 (0.27)	−3.67 (0.54)	−2.97 (0.47)	**−5.94 (26.36)**	2.89 (0.40)
Assertiveness3	2.65 (0.39)	4.13 (0.77)	4.10 (0.80)	3.36 (0.54)	1.81 (0.53)	**2.09 (314.35)**	**2.25 (686.92)**	1.21 (0.31)	−2.85 (0.54)	**−2.98 (314.36)**	**−3.13 (686.93)**	2.14 (0.31)
Assertiveness4	0.74 (0.12)	0.71 (0.64)	0.74 (0.43)	0.77 (0.15)	2.09 (0.61)	**4.07 (25.83)**	**5.49 (39.59)**	1.44 (0.42)	−1.60 (0.62)	**−3.74 (27.82)**	**−5.23 (40.62)**	0.98 (0.37)
Assertiveness5	2.47 (0.38)	4.17 (0.86)	4.19 (1.01)	3.20 (0.57)	−2.73 (0.41)	**−2.92 (28.05)**	**−2.95 (54.15)**	−2.11 (0.24)	−1.23 (0.42)	**−1.72 (28.09)**	**−1.75 (54.24)**	0.80 (0.22)
Assertiveness6	2.32 (0.35)	3.74 (0.56)	4.20 (0.74)	3.23 (0.65)	−2.37 (0.44)	−2.59 (3.22)	−1.81 (0.17)	−1.73 (0.18)	−1.39 (0.44)	−1.77 (3.22)	−1.02 (0.16)	0.91 (0.15)
Assertiveness7	3.23 (0.41)	**7.42 (2.23)**	**7.68 (2.34)**	**4.80 (0.96)**	−2.61 (0.43)	**−2.65 (82.96)**	**−2.64 (201.70)**	−1.97 (0.23)	−1.65 (0.44)	**−1.84 (82.96)**	**−1.85 (201.70)**	1.14 (0.21)
Assertiveness8	2.55 (0.40)	4.28 (0.88)	4.44 (0.95)	3.39 (0.60)	−2.38 (0.40)	−2.00 (0.24)	−1.86 (0.16)	−1.85 (0.17)	−1.29 (0.40)	−1.10 (0.23)	−0.97 (0.16)	0.90 (0.15)
Assertiveness9	1.13 (0.22)	1.32 (0.28)	1.34 (0.28)	1.34 (0.28)	−0.06 (0.14)	−0.02 (0.11)	−0.02 (0.11)	−0.06 (0.12)	−1.37 (0.14)	−1.20 (0.11)	−1.20 (0.11)	1.23 (0.11)
Assertiveness10	1.09 (0.20)	1.30 (0.28)	1.30 (0.28)	1.25 (0.26)	−0.05 (0.15)	−0.01 (0.11)	−0.01 (0.11)	−0.05 (0.13)	−1.23 (0.13)	−1.08 (0.11)	−1.08 (0.11)	1.11 (0.11)
Assertiveness11	1.44 (0.29)	1.88 (0.40)	1.88 (0.39)	1.54 (0.35)	−0.41 (0.12)	−0.34 (0.08)	−0.34 (0.08)	−0.37 (0.11)	−1.36 (0.12)	−1.15 (0.09)	−1.15 (0.09)	1.24 (0.11)

*Note.* The numbers highlighted in bold are the extreme item parameter estimates and huge standard errors.

## Data Availability

Data are available at https://osf.io/4cr6d/ (accessed on 8 June 2023).

## Supplementary Materials

Supplementary Material including appendices, R code, and data is available at https://osf.io/4cr6d/ (accessed on 8 June 2023).
